# GanDouLing promotes proliferation and differentiation of neural stem cells in the mouse model of Wilson’s disease

**DOI:** 10.1042/BSR20202717

**Published:** 2021-01-05

**Authors:** Ting Dong, Ming-cai Wu, Lu-lu Tang, Hai-lin Jiang, Ping Zhou, Chun-jun Kuang, Li-wei Tian, Wen-ming Yang

**Affiliations:** 1 Department of Neurology, The First Affiliated Hospital of Anhui University of Chinese Medicine, Hefei, Anhui, China; 2 Key Laboratory of Xin’an Medicine (Anhui University of Chinese Medicine) Ministry of Education, Hefei, Anhui, China; 3 Department of Biochemistry and Molecular Biology, Wannan Medical College, Wuhu, Anhui, China; 4 Anhui University of Chinese Medicine, Hefei, Anhui, China

**Keywords:** Apoptosis, GanDouLing, Neurogenesis, Wilson's disease

## Abstract

Wilson’s disease (WD) is an autosomal recessive disease caused by mutation of the ATPase copper transporting β (*ATP7B*) gene, resulting in abnormal copper metabolism. We aimed to investigate the protective effect of GanDouLing (GDL) on neural stem cell (NSC) function in a mouse model of WD. NSCs were treated with different concentrations of GDL alone or in combination with penicillamine, following which we evaluated cellular growth, apoptosis, and differentiation. Nuclear factor E2-related factor 2 (Nrf2) pathway and NOD-like receptor family pyrin domain-containing 3 (NLRP3) inflammasome activation were analyzed via Western blotting. Treatment with GDL alone or in combination with penicillamine significantly increased proliferation and inhibited apoptosis of NSCs in a dose-dependent manner. In addition, GDL treatment remarkably promoted differentiation of NSCs. Consistently, levels of class III β-tubulin (Tuj1) and microtubule-associated protein 2 (MAP2) were significantly elevated, whereas glial fibrillary acidic protein (GFAP) levels were obviously suppressed in the presence of GDL or penicillamine. *In vivo* assays confirmed that GDL increased the ratio of Ki67^+^, Tuj1^+^, and MAP2^+^ cells and suppressed apoptosis in the hippocampal region in WD mice. Behavioral assays revealed that both GDL and penicillamine improved memory ability in WD models. Mechanistically, GDL treatment led to activation of Nrf2 signaling and suppression of the NLRP3 inflammasome in WD mice. Notably, inhibition of Nrf2 signaling reversed the protective effects of GDL on hippocampal NSCs. Collectively, these findings demonstrate that GDL exerts a protective effect on NSCs and promotes neurogenesis by targeting Nrf2 signaling and the NLRP3 inflammasome in WD.

## Introduction

Wilson’s disease (WD) is a rare autosomal recessive disorder of copper metabolism that manifests as copper poisoning in various tissues and organs, especially the liver, brain, cornea, and kidneys [1]. The global prevalence of WD is between 1 in 5000 and 1 in 30000. The genetics of WD is complex, with more than 450 disease-causing mutations identified. Trans-membrane ATPase copper transporting β (*ATP7B*) in hepatocytes plays a predominant role in the pathogenesis of WD. An absence of functional ATP7B protein results in decreased hepatocellular excretion of copper into bile. Clinical manifestations of WD vary from an asymptomatic state to acute hepatic failure, chronic liver disease, and neurological symptoms. Early diagnosis and treatment are crucial for effective control of the disease, as life-threatening complications can occur in patients with poor prognosis [2,3]. Thus, the need to develop novel therapeutic drugs and identify the mechanisms underlying their efficacy in WD remains urgent.

Currently, penicillamine is widely used in the treatment of WD due to its low cost and considerable efficacy. However, its application has been associated with many adverse reactions including fever, gastrointestinal reactions, systemic lupus erythematosus, and others [4]. Chinese herbal medicine (CHM) has gained increasing acceptance in clinical settings due to its diverse biological effects, such as anti-oxidant, anti-inflammatory, and apoptosis-suppressing actions. Pharmacological investigations have demonstrated that traditional Chinese medicine (TCM) can improve urinary copper excretion, alleviate hepatic fibrosis, and protect the brain, liver, and kidneys [3–5]. GanDouLing (GDL), a Chinese medicinal herb, has been shown to play critical roles in treating blood stasis, invigorating blood circulation, and promoting copper excretion in the liver and gallbladder [6]. Previously, our team demonstrated that GDL combined with penicillamine improves cerebrovascular injury via the PERK/eIF2α/CHOP endoplasmic reticulum stress pathway in WD model animals [6]. In the present study, we investigated the effect of GDL on neurological function in animal models of WD, as well as the mechanisms associated with this effect.

## Materials and methods

### Animals

Forty-eight specific pathogen-free (SPF)-grade female toxic milk (TX) C3He-Atp7btx-J/J mice weighing 20–25 g were obtained from the Jackson Laboratory and raised in the animal center of the Ministry Key Laboratory of Anhui University of Chinese Medicine. Animals were housed in an environment with controlled humidity (50–70%) at room temperature. The animals were fed in isolation cages with independent air supplies and were given free access to food and water. All protocols involving animals were reviewed and approved by the Institutional Animal Care and Use Committee of the First Affiliated Hospital of Anhui University of Chinese Medicine (approval number: **AHAU2018008**). All procedures performed in the present study were in accordance with the ethical standards of the institutional research committee and the 1964 Helsinki Declaration and its later amendments or comparable ethical standards.

### Neural stem cell primary cultures

Wild-type newborn C57BL/6 mice were killed using 2% sodium pentobarbital (50 mg/kg), following which whole brains were removed and washed in D-Hanks buffer. Hippocampal tissues were separated and gently rinsed in D-Hanks buffer. The hippocampal tissues were then cut into small pieces, digested in a collagenase solution and further with trypsin, re-suspended in neurobasal culture medium (Gibco, U.S.A.), and inoculated into a 25-ml flask. Cells were cultured in proliferation medium [DMEM/F12 (1:1) supplemented with 0.2% heparin, 1× B27 supplement (Gibco), 20 ng/ml mouse EGF, 10 ng/ml mouse bFGF, Pen/Strep, and l-glutamine] at 37°C in a chamber with 5% CO_2_. Proliferation medium was changed every other day, and cells were passed every 4–5 days when flasks were 80–90% confluent. Cells were then seeded into 96-well plates at a density of 10^3^ cells per well for subsequent experiments.

### GDL preparation

GDL was produced by the First Affiliated Hospital of Anhui University of Chinese Medicine: the active ingredients of each herb were extracted with 65% ethanol and then combined with extracted filtrate. The filtrate combinations were baked into dry paste at the right temperature, following which starch was added and packed into the GDL troche, as previously described [6]. Then, GDL was diluted in ethanol, and dose–responses were determined at concentrations of 1, 5, 10, and 25 μg/ml. A concentration of 10 μg/ml was used for single-dose GDL administration. Then, 5 μm ML385 (purity > 98%; Sigma) combined with GDL was applied. For the *in vivo* study, GDL was intragastrically administered at a dose of 0.486 g/kg (body weight) as previously described [6]. Six mice were used in each group for the *in vivo* assays.

### Methyl thiazolyl tetrazolium assay

Based on the manufacturer’s protocol, methyl thiazolyl tetrazolium (MTT) assays were performed to determine the effect of different doses of GDL on neural stem cell (NSC) proliferation. Briefly, cells were seeded on to 96-well plates at a density of 10^3^ cells per well, and the cell viability in each well was determined by adding MTT solution. After further incubation at 37°C for 2 h, absorbance was measured using an enzyme-linked immunosorbent assay (ELISA) reader at a wavelength of 490 nm. At least three independent experiments with three replicates were performed.

### Terminal deoxynucleotidyl transferase d-UTP nick-end labeling

Terminal deoxynucleotidyl transferase d-UTP nick-end labeling (TUNEL) assays were performed to examine cellular apoptosis using an *in situ* cell death detection kit (Roche, Mannheim, Germany), in accordance with the manufacturer’s instructions. Briefly, cells were fixed in ethanol/acetic acid (2:1), incubated in 3% H_2_O_2_, permeabilized with 0.5% Triton X-100, and then incubated in the TUNEL reaction mixture. Sections were rinsed, stained with 4′,6-diamidino-2-phenylindole (DAPI), and observed under a fluorescence microscope.

### Bromodeoxyuridine assay

For the bromodeoxyuridine (BrdU) assay, cells were fixed in 4% paraformaldehyde for 20 min. After washing three times with phosphate-buffered saline (PBS), cells were incubated with primary anti-BrdU antibody (Sigma–Aldrich, U.S.A.) at 4°C overnight and with secondary antibody at room temperature for 1 h. Images were taken under a confocal microscope.

### Animal models of WD

Forty-eight female TX mice were divided into a control group, a Wilson group, a GDL group, a penicillamine group, and a GDL-penicillamine group. Mice in the GDL and penicillamine groups received intragastric GDL or penicillamine, respectively, as previously described [6]. Mice in the GDL-penicillamine group were treated with both GDL and penicillamine via intragastric administration. The Wilson and control groups were treated via an intragastric administration of an equivalent volume of 0.9% saline everyday. All animals were killed by intraperitoneal injection of 2% sodium pentobarbital (50 mg/kg).

### Quantitative real-time polymerase chain reaction

Total RNA was extracted using TRIzol (Invitrogen, U.S.A.), in accordance with the manufacturer’s instructions. Briefly, 1 μg RNA was used to synthesize cDNA using M-MLV Reverse Transcriptase (Promega, Fitchburg, WI). The PCR was performed using the SYBR Green PCR Master Mix (Applied Biosystems, Foster City, CA). The primer sequences used were as follows: Tuj1: 5′-GGCCAAGGGTCACTACACG-3′ (forward) and 5′-GCAGTCGCAGTTTTCACACTC-3′ (reverse); glial fibrillary acidic protein (GFAP): 5′-CTGCGGCTCGATCAACTCA-3′ (forward) and 5′-TCCAGCGACTCAATCTTCCTC-3′ (reverse); microtubule-associated protein 2 (MAP2): 5′-CTCAGCACCGCTAACAGAGG-3′ (forward) and 5′-CATTGGCGCTTCGGACAAG-3′ (reverse); nuclear factor E2-related factor 2(Nrf2): 5′-TCAGCGACGGAAAGAGTATGA-3′ (forward) and 5′-CCACTGGTTTCTGACTGGATGT-3′ (reverse); β-actin: 5′-CTGTATGCCTCTGGTCGTAC-3′ (forward) and 5′-TGATGTCACGCACGATTTCC-3′ (reverse). PCR consisted of an initial denaturing step (95°C, 5 min) followed by 40 cycles of denaturing (95°C, 15 s), annealing (60°C, 15 s), and extension (72°C, 45 s). The relative expression levels of each gene were determined using the comparative cycle threshold (*C*_t_) method by calculating 2^(−ΔΔ*C*_t_)^. β-actin was used as the endogenous control.

### Western blotting

The samples from each group were separated using sodium dodecyl sulfate (SDS)/polyacrylamide gels, following which they were transferred on to nitrocellulose membranes (Merck Millipore; Darmstadt, Germany). After blocking with 5% skim milk, the membranes were incubated with primary antibodies against nuclear Nrf2, NAD(P)H:quinone oxidoreductase 1 (NQO1), heme oxygenase-1 (HO-1), catalytic subunit of γ-glutamylcysteine ligase (GCLC), NOD-like receptor family pyrin domain-containing 3 (NLRP3), caspase-1, interleukin (IL), and β-actin (Santa Cruz Biotechnology, Santa Cruz, CA, U.S.A.) at 4°C overnight and then with the secondary antibodies for 2 h at room temperature. Images were obtained using electrochemiluminescence (ECL).

### Statistical analysis

Data are expressed as the mean ± standard deviation. All data were analyzed using Student’s *t* test or one-way analyses of variance, followed by Dunnett’s *t* test for comparisons among multiple groups. *P*-values <0.05 were considered statistically significant.

## Results

### Effects of GDL treatment on NSC viability and apoptosis *in vitro*

**Figure 1:**
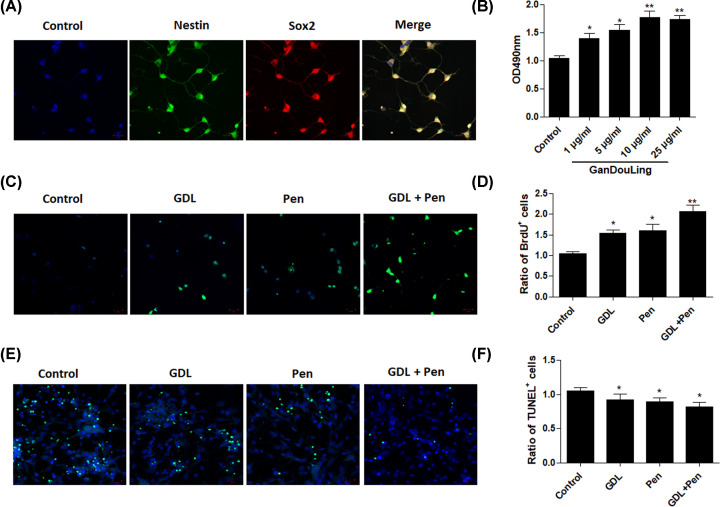
Effects of GDL treatment on NSC viability and apoptosis *in vitro* Immunostaining and identification with Nestin and Sox2 in NSCs (**A**). MTT assay was performed after treatment with different concentrations of GDL (**B**). NSCs were incubated with BrdU (**C,D**) and TUNEL (**E,F**) to confirm the effect of GDL on cell proliferation and apoptosis. *P<0.05, **P<0.01, compared with control.

We first explored whether GDL affects the proliferation of NSCs *in vitro*. NSCs were identified based on positive staining for Nestin (an NSC marker) and Sox2 (a stem cell marker) (Figure 1A). To evaluate the effect of GDL treatment on NSC viability, MTT assays were performed after treatment with different concentrations of GDL (1, 5, 10, and 25 μg/ml). GDL treatment at different concentrations gradually increased NSC viability in a dose-dependent manner (Figure 1B). NSCs were incubated with BrdU to confirm the effect of GDL on cell proliferation. The data showed that treatment with GDL or penicillamine alone or in combination significantly increased the number of BrdU+ cells (Figure 1C,D). In addition, TUNEL assays were performed to evaluate the effect of GDL treatment on apoptosis of NSCs. Relative to those observed in the control group, rates of apoptosis were obviously decreased in NSCs treated with GDL or penicillamine alone or in combination (Figure 1E,F). Collectively, these results indicated that GDL increases cell viability and inhibits apoptosis of NSCs.

### Effects of GDL treatment on the differentiation of NSCs *in vitro*

**Figure 2:**
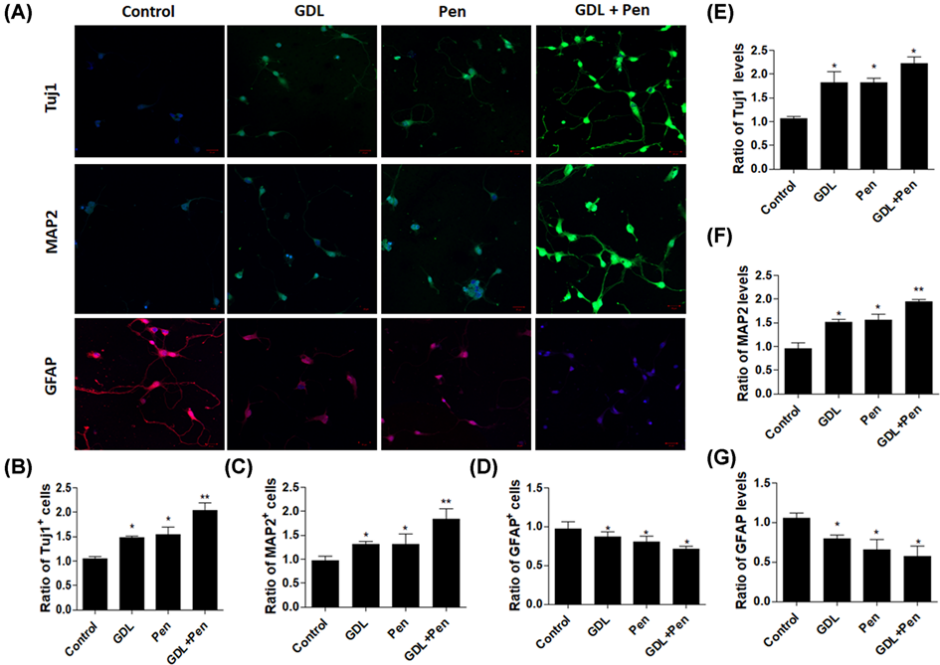
Effects of GDL treatment on the differentiation of NSCs *in vitro* Immunostaining with Tuj1, MAP2, GFAP in NSCs treated with GDL, penicillamine, and GDL plus penicillamine (**A**–**D**). In addition, mRNA levels of Tuj1, MAP2, and GFAP in NSCs treated with GDL, penicillamine, and GDL plus penicillamine were determined using quantitative real-time polymerase chain reaction (**E**–**G**). *P<0.05, **P<0.01, compared with control.

Since GDL exerts a positive effect on NSC proliferation, we investigated whether GDL also affects the differentiation of NSCs. In the presence of GDL or penicillamine, we observed significant increases in the number of Tuj1+ and MAP2+ cells generated from neurospheres. In addition, combined treatment with GDL and penicillamine further increased the ratio of Tuj1+ and MAP2+ cells generated from neurospheres. In contrast, we observed significant decreases in GFAP-positive (GFAP+) glial cells in MLB- or penicillamine-treated NSCs. Combined treatment with GDL and penicillamine further decreased the ratio of GFAP+ glial cells (Figure 2A–D). Furthermore, real-time PCR was performed to determine mRNA levels of Tuj1, MAP2, and GFAP in NSCs. Consequently, mRNA levels of Tuj1 and MAP2 were significantly elevated, whereas that of GFAP was obviously suppressed, in the presence of GDL or penicillamine. Combined treatment with GDL and penicillamine further promoted expression of Tuj1 and MAP2 and inhibited GFAP (Figure 2E–G). Collectively, these data demonstrated that treatment with GDL can induce the differentiation of NSCs toward neurons.

### Effects of GDL treatment on local neurogenesis *in vivo*

**Figure 3:**
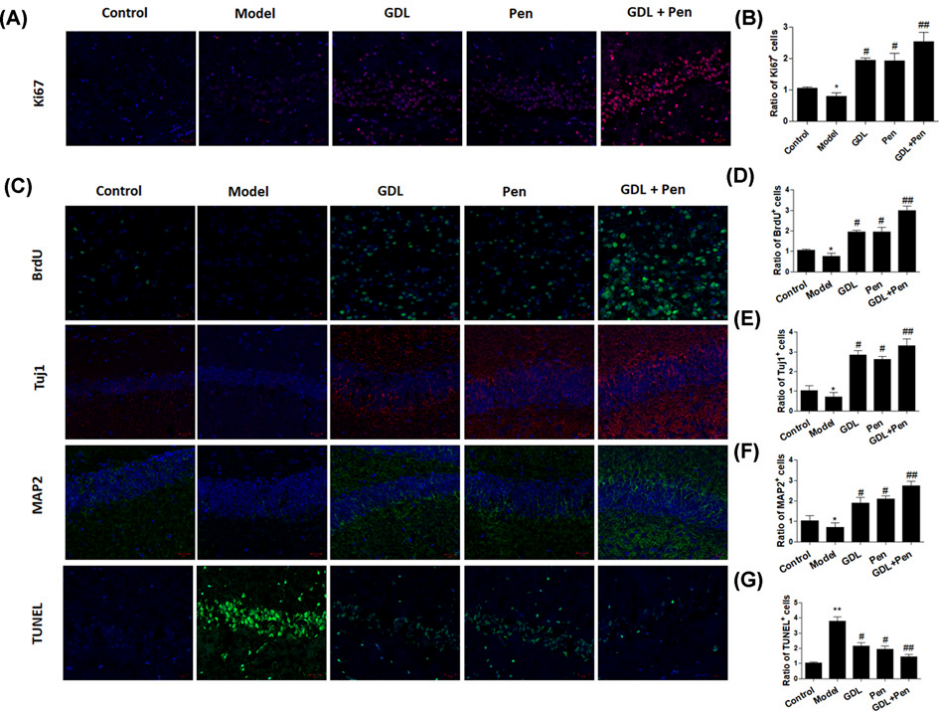
Effects of GDL treatment on the local neurogenesis *in vivo* WD mice were treated with intragastric administration with GDL, penicillamine, and GDL plus penicillamine. Ratios of Ki67+ (**A,B**), BrdU+ (**C,D**), Tuj1+ (**C,E**), MAP2+ (**C,F**), and TUNEL+ (**C,G**) cells were determined in the hippocampal regions in WD mice. *P<0.05, **P<0.01, compared with control; #*P*<0.05, ##*P*<0.01, compared with model.

We explored the *in vivo* effect of GDL by treating mice with an intragastric administration of GDL or penicillamine alone or in combination. Our data indicated that administration of GDL and penicillamine both increased the ratio of Ki67+ cells in the hippocampal region (Figure 3A,B). In contrast, the apoptosis rate in the hippocampal region was obviously increased in model mice, while treatment with GDL or penicillamine alone or in combination significantly suppressed apoptosis in the hippocampal region (Figure 3C,D). Moreover, the ratios of Tuj1+ and MAP2+ cells were significantly suppressed in the model group but dramatically elevated in the hippocampal region in mice treated with GDL, penicillamine, or both (Figure 3C,E–G). Collectively, these data demonstrate the positive effect of GDL on neurogenesis in WD mice.

### Effects of GDL treatment on symptoms in WD animals

**Figure 4:**
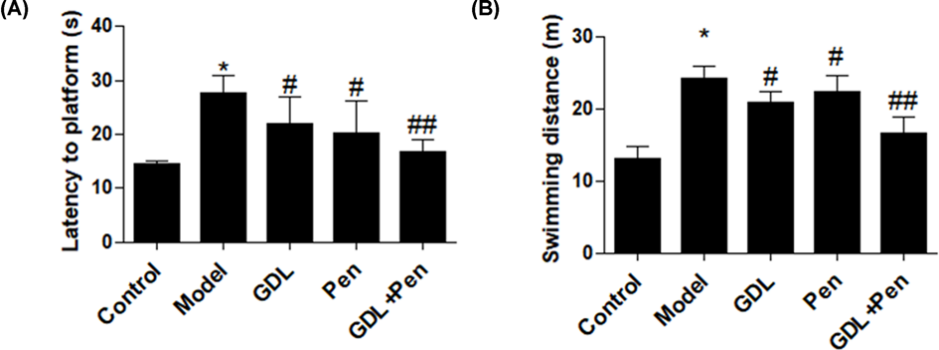
Effects of GDL treatment on symptoms in WD animals WD mice were treated by intragastric administration with GDL, penicillamine, and GDL plus penicillamine. The latency to platform and swimming distance were determined in each group. *P<0.05, compared with control; #*P*<0.05, ##*P*<0.01, compared with model.

To determine the potential clinical applicability of GDL, we investigated whether GDL could alleviate the symptoms of WD. Latency to the platform and swimming distance in the Morris water maze test were significantly greater in WD mice than in control mice, whereas treatment with GDL or penicillamine decreased the swimming distance (Figure 4A,B). GDL combined with penicillamine further improved latency to the platform and swimming distance. Taken together, these findings suggest that GDL treatment can promote neurogenesis and improve memory ability in WD models.

### Effects of GDL treatment on the Nrf2 and NLPR3 pathways in WD models

**Figure 5:**
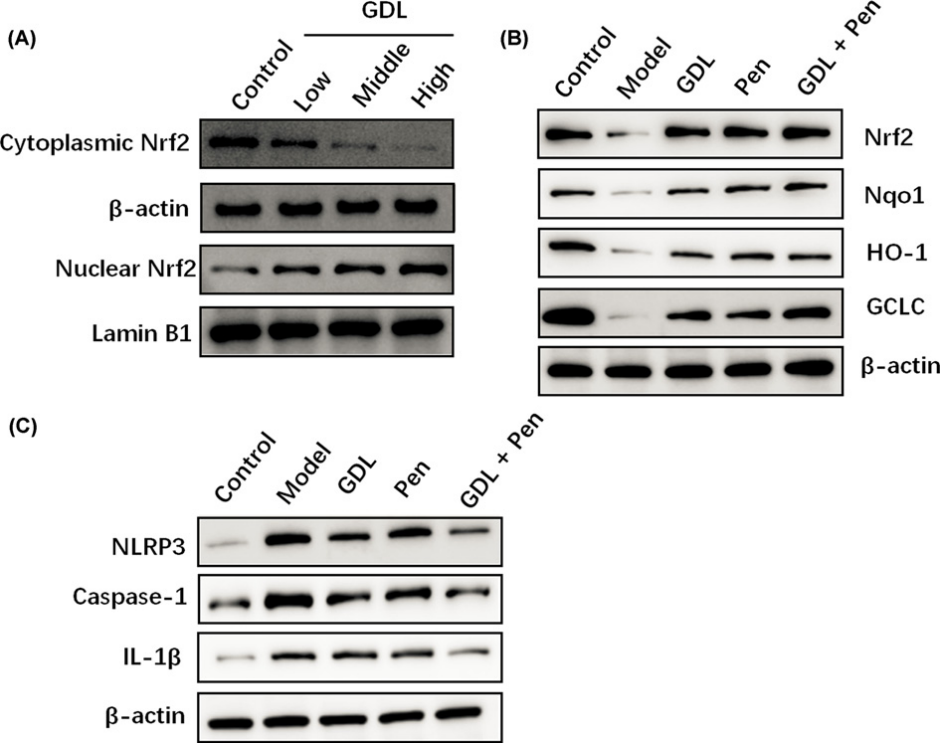
Effects of GDL treatment on Nrf2 pathway in WD mice Western blotting assay was performed to determine the cytoplasmic and nuclear Nrf2 levels after treatment with different concentrations of GDL in NSCs (**A**). WD mice were treated by intragastric administration with GDL, penicillamine, and GDL plus penicillamine. Expression of Nrf2 and its downstream targets (GCLC, HO-1, and NQO1) in different groups were detected by Western blotting (**B**). Protein expression of NLPR3, Caspase-1, and IL-1β in different groups was detected by Western blotting (**C**).

Given that the Nrf2 signaling pathway plays an important role in hippocampal neurogenesis, we investigated whether Nrf2 signals mediate the protective effects of GDL in WD mice. Western blotting revealed that GDL treatment decreased cytoplasmic Nrf2 levels and increased nuclear Nrf2 expression in NSCs (Figure 5A). In addition, the expression level of Nrf2 protein in the hippocampal region was markedly decreased in WD mice but significantly increased in mice treated with GDL or penicillamine (Figure 5B). We also assessed protein levels of downstream targets of Nrf2 including GCLC, HO-1, and NQO1. Consequently, the data showed that GDL or penicillamine treatment promoted the expression of all three proteins in the hippocampal region in WD models. Strikingly, GDL combined with penicillamine led to further increases in the activation of the Nrf2 signaling pathway in WD mice (Figure 5B). Furthermore, we explored whether GDL treatment inhibits NLRP3 inflammasome activation in WD mice. Western blotting results indicated that the expression levels of NLPR3, caspase-1, and IL-1β were significantly elevated in WD mice but dramatically decreased in the GDL and penicillamine groups (Figure 5C). Taken together, these results demonstrated that GDL promotes neurogenesis and alleviates symptoms of WD by activating Nrf2 and suppressing the NLPR3 inflammasome in animal models.

### Effects of ML385-induced suppression of Nrf2 on the neuroprotective actions of GDL

**Figure 6:**
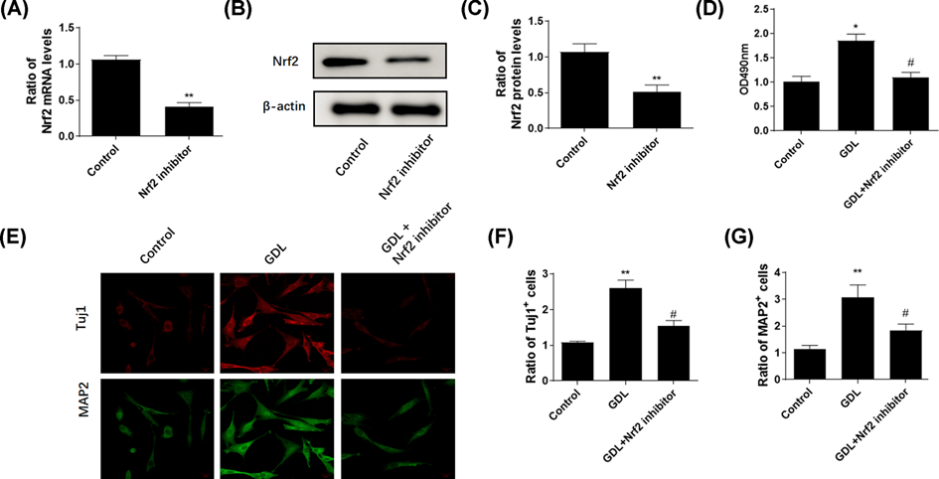
ML385 reversed the protective effects of GDL Real-time PCR (**A**) and Western blotting (**B,C**) assays were performed to determine the mRNA and protein levels of Nrf2 after treatment with GDL alone or in combination with Nrf2 inhibitor, ML385. MTT assay was performed after treatment with GDL alone or combined with ML385 (**D**). Immunostaining with Tuj1 and MAP2 in NSCs treated with GDL alone or combined with ML385 (**E**). Ratios of Tuj1+ (**F**) and MAP2+ NSCs (**G**). *P<0.05, **P<0.01, ^#^*P*<0.05, compared with GDL group.

To further ascertain the role of the Nrf2 pathway in the effect of GDL, we used ML385 to suppress the Nrf2 pathway. As expected, the mRNA and protein levels of Nrf2 were significantly inhibited in ML385-treated cells (Figure 6A–C). Consequently, treatment with ML385 reversed the protective effect of GDL on the viability of NSCs (Figure 6D). Furthermore, ML385 decreased the ratio of Tuj1+ and MAP2+ cells and thus delayed the differentiation of NSCs (Figure 6E–G). Collectively, these findings demonstrated that inhibition of Nrf2 by ML385 can reverse the neuroprotective effects of GDL in NSCs.

## Discussion

In the present study, we investigated the effects of GDL on neurological function and neurogenesis in animal models of WD, as well as the mechanisms associated with these effects. Our findings indicated that GDL promoted cell viability and suppressed both apoptosis and oxidative stress in NSCs. Mechanistically, our findings suggested that GDL protected against copper-induced NSC injury by enhancing the Nrf2 pathway and inhibiting NLRP3 inflammasome activation.

Several previous studies have highlighted defects of the central nervous system (CNS) in patients with WD [7,8]. A positron emission tomography (PET) study by Hawkins et al. demonstrated decreased perfusion in the cerebral cortex of patients with WD [9]. Similarly, Smith et al. reported decreased cerebral blood flow in the bilateral thalamus in patients with WD [10]. Furthermore, Chen et al. observed that WD induced cerebrovascular injury in a mouse model of the disease, while GDL alone or in combination with penicillamine improved such injury [6].

NSCs are multipotent stem cells able to self-renew and generate immature and differentiated cell populations [11,12]. Accumulating evidence indicates that NSCs play a critical role in cognitive functions and memory ability in several animal models of human diseases of cognition, such as Parkinson’s disease and Alzheimer’s disease [13]. In our study, NSCs were treated with different doses of GDL in order to evaluate its neuroprotective effects. Consequently, GDL treatment alone or in combination with penicillamine significantly increased proliferation, inhibited apoptosis, and promoted differentiation of NSCs, suggesting a neuroprotective effect of GDL in WD.

As previously mentioned, NSCs are multipotent cells with the capacity for self-renewal, and they can differentiate into both neurons and glial cells. Evidence suggests that TCM can promote differentiation and increase local neurogenesis. These beneficial effects of constituents from herbal drugs have been observed in mouse models of CNS-related diseases, including Parkinson’s disease and Alzheimer’s disease [14,15]. In our study, administration of GDL or penicillamine increased proliferation and suppressed apoptosis in the hippocampal region. Moreover, ratios of Tuj1+ and MAP2+ cells in the hippocampal region were significantly elevated in GDL- or penicillamine-treated mice, suggesting the beneficial role of GDL on neurogenesis *in vivo*. Indeed, behavioral assays revealed that GDL and penicillamine both improved memory ability in WD models, as indicated by decreases in latency to the platform and swimming distance.

Nrf2 is considered an important endogenous regulator of various physiological and pathological events. Activation of the Nrf2 signaling pathway leads to elevated expression of its downstream targets, including HO-1, GCLC, and NQO1. Previous studies have demonstrated that Nrf2 is involved in regulating the self-renewal and differentiation of NSCs during embryogenesis [16]. Newborn Nrf2 knockout (Nrf2^−/−^) mice exhibit decreased neuronal numbers and attenuated NSC function [17]. In contrast, activation of Nrf2/HO-1 by garcinone D, a natural xanthone, may promote the proliferation of endogenous NSCs [18]. Inflammatory cytokines such as IL-1β play important roles in the development of NSC injury. The NLRP3 inflammasome is responsible for the processing and secretion of mature IL-1β [19,20]. As expected, GDL treatment decreased cytoplasmic Nrf2 levels and increased nuclear Nrf2 expression in NSCs. Additionally, GDL treatment increased protein levels of Nrf2 and its downstream antioxidant genes (GCLC, HO-1, and NQO1) in the hippocampal region in WD mice, suggesting that GDL exerts a neuroprotective effect in WD via the Nrf2 signaling pathway. Inhibition of Nrf2 signaling reversed the protective effects of GDL on hippocampal NSCs. Moreover, GDL and penicillamine treatment both decreased levels of NLPR3, caspase-1, and IL-1β in the hippocampal region in WD models. Strikingly, GDL combined with penicillamine exerted a stronger effect on these molecular targets. These findings suggest that GDL exerts neuroprotective effects by regulating Nrf2 signaling during WD pathogenesis.

In conclusion, the present study demonstrated that GDL promotes proliferation and differentiation of NSCs *in vitro* and increases neurogenesis *in vivo* in WD models. Mechanistically, activation of Nrf2 signaling and suppression of the NLRP3 inflammasome may be responsible for the neuroprotective effects of GDL in WD. These findings provide novel insight into the molecular mechanisms underlying the pathogenesis of WD and support the potential application of GDL in patients with this disease.

## Data Availability

The raw data supporting the conclusions of this manuscript will be made available by the authors, without undue reservation, to any qualified researcher.

## Abbreviations

*ATP7B*: ATPase copper transporting β
bFGF: basic fibroblast growth factor
BrdU: bromodeoxyuridine
CHOP: C/EBP-homologous protein
CNS: central nervous system
*C*_t_: cycle threshold
EGF: epidermal growth factor
eIF2α: eukaryotic initiation factor 2α
GCLC: catalytic subunit of γ-glutamylcysteine ligase
GDL: GanDouLing
GFAP: glial fibrillary acidic protein
HO-1: heme oxygenase-1
IL: interleukin
MAP2: microtubule-associated protein 2
MTT: methyl thiazolyl tetrazolium
NLRP3: NOD-like receptor family pyrin domain-containing 3
NQO1: Nrf2, NAD(P)H:quinone oxidoreductase 1
Nrf2: nuclear factor E2-related factor 2
NSC: neural stem cell
Pen/Strep: Penicillin Streptomycin
PERK: Protein kinase R (PKR)-like endoplasmic reticulum kinase
qRT-PCR: quantitative real-time polymerase chain reaction
TCM: traditional Chinese medicine
TUNEL: terminal deoxynucleotidyl transferase d-UTP nick-end labeling
TX: toxic milk
WD: Wilson’s disease
